# 

**Published:** 1887-10

**Authors:** 


**Vol. XLI.** **OCTOBER 1887.** **No. 10.**

### THE DENTAL REGISTER

LIBRARY
INDEXED
S.G.O.

A MONTHLY JOURNAL,

DEVOTED TO THE INTERESTS OF THE PROFESSION.

EDITED BY

J. TAFT, D.D.S.

**PUBLISHED BY**

SAM'L A. CROCKER & CO.,

SUCCESSORS TO SPENCER & CROCKER,

OHIO DENTAL AND SURGICAL DEPOT,

Nos. 117, 119, 121 WEST FIFTH STREET, CINCINNATI, OHIO.

**TERMS:—$2.00 per Annum, in Advance. Single Copies, 25 cts.**

